# Prevalence of alexithymia and its association with anxiety and depression in a sample of Greek chronic obstructive pulmonary disease (COPD) outpatients

**DOI:** 10.1186/1744-859X-9-16

**Published:** 2010-04-14

**Authors:** Athanasios Tselebis, Epaminondas Kosmas, Dionisios Bratis, Georgios Moussas, Athanasios Karkanias, Ioannis Ilias, Nikolaos Siafakas, Alexandros Vgontzas, Nikolaos Tzanakis

**Affiliations:** 1 Psychiatric Department, Sotiria General Hospital of Chest Diseases, Athens, Greece; 2 Pulmonary Rehabilitation Centre, Sotiria General Hospital of Chest Diseases, Athens, Greece; 3 Endocrine Department, Elena Venizelou Hospital, Athens, Greece; 4 Department of Thoracic Medicine, University of Crete, Medical School, Heraklion, Greece; 5 Psychiatric Department, University of Crete, Medical School, Heraklion, Greece; 6 Departement of Social Medicine, Laboratory of Epidemiology, University of Crete, Medical School, Heraklion, Greece

## Abstract

**Background:**

Chronic obstructive pulmonary disease (COPD) is a major health problem, especially in adults over 40 years of age, and has a great social and economic impact. The psychological morbidity of COPD patients with regard to anxiety and depressive symptoms has been extensively studied in the past. However, few studies have investigated the prevalence of alexithymia in these patients, as well as its association with this comorbidity. Based on this fact, we studied the prevalence of alexithymia and its association with anxiety and depressive symptoms in COPD outpatients.

**Methods:**

The present study included 167, randomly selected, outpatients diagnosed with COPD. Alexithymia, anxiety and depression were assessed using the Toronto Alexithymia Scale (TAS-20), Spielberger Trait Anxiety Inventory (STAI), and Beck Depression Inventory (BDI), respectively.

**Results:**

The mean BDI score was 12.88 (SD: 7.7), mean STAI score 41.8 (SD: 11.0) and mean TAS-20 score 48.2 (SD: 11.5). No differences were observed between genders regarding age and alexithymia (t test *P *> 0.05), while female patients presented higher depression and trait anxiety scores than males (t test *P *< 0.05). Clinically significant levels of anxiety were present in 37.1% of men, and in 45.7% of women. The mean depression score was also higher than the corresponding mean score in the general population (one-sample t test *P *< 0.01), while 27.7% and 30.5% of the sample presented mild and moderate to severe depression, respectively. Finally, a strong correlation was observed between alexithymia, depression and anxiety.

**Conclusions:**

This study confirms the high prevalence of anxiety and depression symptoms in Greek outpatients with COPD. The prevalence of alexithymia in COPD patients, contrary to what has been observed in patients with other chronic respiratory diseases, seem to be lower. However, we observed a strong association between alexithymia, depression and anxiety levels. This observation suggests that alexithymia should be taken into consideration when drafting specific psychotherapeutic interventions for these patients.

## Background

Chronic obstructive pulmonary disease (COPD) is one of the leading causes of mortality and morbidity worldwide. The disease is very common especially in smoker adults over 40 years of age and has a considerable social and economic impact [1]. In the USA it is the fourth highest ranked condition leading to chronic morbidity and mortality and, according to the World Health Organization (WHO), it is expected to rank fifth in the year 2020 for burden of disease worldwide [2,3]. The disease is characterised by airflow obstruction that is not fully reversible; this airflow limitation is usually progressive and is associated with an abnormal inflammatory response of the lungs to noxious particles or gases, primarily caused by cigarette smoking [1].

With regard to Greece, studies from the 1980s pointed to this disease being a public health problem [4]. At the same time, COPD's effect on the psychological status of patients was recognised [5].

However, the relevant psychological status studies have mainly been focused on the prevalence of anxiety [6-10] and depression, which often appear together in these patients [11-15]. The prevalence of depression among outpatients with COPD is substantially greater than lifetime rates in the general population (ranging between 10% and 42% in the former, compared to approximately 5% in the latter). Correspondingly, the prevalence of anxiety varies from 10% to 19% [15], a percentage that is higher than the 15% that is reported in the general population [16,17]. Regarding patients who have recently recovered from an acute exacerbation of COPD, the prevalence of depression is even higher and ranges between 19.4% and 50% [15]. The same is true concerning anxiety, with the percentage ranges between 9.3% and 58% [15].

Both depression and anxiety are significantly associated with decreased functional status and worse health status when compared to those of patients without psychological symptoms, even after controlling for the effects of overall health status [13,17-19]. Higher predominance of depression and anxiety was observed in patients with COPD compared to patients that suffered from other chronic respiratory disorders, such as bronchial asthma and tuberculosis [17].

Alexithymia indicates 'lack of words for emotions' [20]. This term was first used in the 1970s, when Nemiah and Sifneos, evaluating psychiatric interviews of patients with classic psychosomatic ailments, found that most of these patients had great difficulty in describing their feelings verbally as well as limited capacity to fantasise [21]. Sifneos [20], in an effort to describe these symptoms under a coherent term coined the phrase alexithymia (loosely based on ancient Greek, from αλέξε = deflect + θυμικο = the emotional moiety of the soul); thus it literally implies a deflection of emotions. In general, subjects with alexithymia usually complain of somatic symptoms, regardless of their somatic ailment. At the same time they have great difficulty in recognising and describing their emotions [20-22]. Although the role of alexithymia and its association with levels of anxiety and depression has already been recognised in other respiratory diseases, such as bronchial asthma [23], few studies have investigated the possibility that alexithymia may also be prevalent in patients suffering from COPD [24-26].

Taking into account the high prevalence of anxiety and depression in patients with COPD [15], as well the reported associations among depression, anxiety, somatic symptoms and alexithymia [27], we studied the prevalence of alexithymia and its association with anxiety and depression in a sample of Greek COPD outpatients.

## Methods

### Sample

From the outpatients list of scheduled appointments at our hospital's clinics (among the largest respiratory disease hospitals in Europe) we selected those to be included in the study with randomisation using a Microsoft Excel (Microsoft, Redmond, WA, USA) algorithm (167 COPD outpatients). No patient refused to participate in the study. Subjects over the age of 80 years and patients diagnosed with other major somatic disorders (such as heart failure, myocardial infarction, cerebrovascular disease, cancer, or severe orthopaedic disorders) or major mental disorders (such as schizophrenia or sentimental disorder) were excluded from the study. The relevant information was obtained from the subjects' medical history and medical record. Age, gender, family status and education years were noted.

### Physical measures

In order to determine the COPD severity of our sample, a spirometric evaluation before and after bronchodilation (200 μg salbutamol) was performed. We followed the Global Initiative for Chronic Obstructive Lung Disease (GOLD) diagnostic criteria, which classify COPD severity (in relation to forced expiratory volume in 1 s (FEV_1_) percentage of predicted) into four stages. Stage I (mild COPD): FEV_1 _> 80% predicted; stage II (moderate COPD): FEV_1 _50% to 80% predicted; stage III (severe COPD): FEV_1 _30% to 50% predicted; and stage IV (very severe COPD): FEV_1 _< 30% predicted [3].

### Psychological measures

Depression was assessed with the Beck Depression Inventory (BDI) [28], which includes 21 items graded from 0 to 3. A high total score in the questionnaire corresponds to the presence of elevated depressive symptomatology [16,17,23,29]. The inventory has been standardised and used in Greek population. The inner coherence reliability (α = 0.84) is high and the retest reliability ranges from 0.48 to 0.86 for clinical groups and 0.60 to 0.90 for non-clinical populations. Its validity in relation to an external criterion for depression (that is, a clinical diagnosis) is considered to be satisfactory [30].

Anxiety was assessed with the Spielberger State Trait Anxiety Inventory (STAI), a widely used anxiety rating scale [31]. It consists of 40 items, each graded from 1 to 4. The scale differentiates anxiety into (a) anxiety caused by a specific condition (state subscale) and (b) anxiety as a more permanent characteristic of personality (trait subscale). The Greek validation of the trait subscale was used in our study, which is considered as having a high inner coherence reliability (α = 0.89) and validity compared to clinical diagnosis [32,33].

Alexithymia was assessed with the Toronto Alexithymia Scale (TAS-20), which includes 20 items, graded from 1 to 5. A high score (> 60) on the scale is consistent with increased presence of alexithymic characteristics [34]. The TAS-20 has been adapted into the Greek language and its reliability is considered to be satisfactory (α = 0.80) [35].

Before replying to the questionnaires used in this study, all the subjects were evaluated by two clinical psychologists in 60 min person-to-person sessions.

Statistical analysis was performed with analysis of variance (ANOVA) and Tukey's *post hoc *tests, Student t test and stepwise multiple linear regression. Statistical significance was set at *P *< 0.05 (corrected where applicable).

The hospital ethics committee approved the study and all participants provided written informed consent. No financial support was necessary.

## Results

The sample included 132 men and 35 women. The mean age of the participants was 65.5 ± 8.2 (men: 65.4 ± 8.1 and women 65.7 ± 8.5), while the mean FEV_1 _percentage of predicted was 41.5 ± 18.7 (Table 1). There were no differences between genders, regarding age, years of education, and FEV_1 _percentage of predicted (ANOVA *P *> 0.05, Table 1). The family status of the study's subjects (77.7% were married, 4.6% were single, 7.7% were widowed and 10% were divorced) was not found to be associated with the studied parameters (one-way ANOVA *P *> 0.005).

**Table 1:** Demographics and baseline characteristics.

Characteristic	Value
Male/female	132/35

Age, years (± SD)	

Male	65 ± 8

Female	66 ± 9

Education, years (± SD)	

Male	11 ± 4

Female	11 ± 5

FEV_1 _(percentage of predicted) (± SD)	

Male	41 ± 19

Female	46 ± 17

Severity (GOLD)	

Mild/moderate/severe/very severe	10/30/75/52

FEV_1 _= forced expiratory volume in 1 s; GOLD = Global Initiative for Chronic Obstructive Lung Disease.

With regard to the severity of COPD according to the GOLD classification scheme, 10 patients had mild disease, 30 moderate disease, 75 severe disease and 52 were at a very severe stage of the disease.

Mean BDI score was 12.88 ± 7.7, mean STAI score was 41.8 ± 11.0 and mean TAS-20 score was 48.2 ± 11.5. No differences were observed between genders, regarding age and alexithymia (TAS-20) (ANOVA *P *> 0.05, Table 2), while female patients presented higher depression (BDI) and trait anxiety (STAI) scores than males (ANOVA *P *< 0.05, Table 2).

**Table 2:** Mean (± SD) scores for Beck Depression Inventory (BDI), Spielberger Trait Anxiety Inventory (STAI) and Toronto Alexithymia Scale (TAS-20).

Category	Value
Depression	

Male (N = 132)	12.2 ± 7.6

Female (N = 35)	15.3 ± 7.9

Total (N = 167)	12.9 ± 7.7

Anxiety	

Male (N = 132)	41.1 ± 10.4

Female (N = 35)	44.7 ± 10.6

Total (N = 167)	41.8 ± 10.5

Alexithymia	

Male (N = 132)	48.0 ± 11.2

Female (N = 35)	49.2 ± 12.8

Total (N = 167)	48.2 ± 11.2

Females had higher trait anxiety and depression scores than males (t test *P *< 0.05, for all comparisons).

Patients with COPD presented the same mean alexithymia score (48.2) as the general population's mean score (49.5) [35] (one-sample t test *P *> 0.05). However, 12% of our sample presented with a score > 60 (Table 3).

**Table 3:** Prevalence of anxiety, alexithymia and depressive symptoms in relation to gender.

	Anxiety (STAI)	Alexithymia (TAS-20)	Mild depression (BDI 10-14)	Moderate to severe depression (BDI ≥ 15)
Male	37.1%	10.6%	25%	30.3%

Female	45.7%	17.1%	20%	54.3%

Total	38.3%	12%	24%	35.3%

BDI = Beck Depression Inventory; STAI = Spielberger Trait Anxiety Inventory; TAS-20 = Toronto Alexithymia Scale.

Men presented higher mean trait anxiety levels (ANOVA *P *< 0.01) than the corresponding level (34.54) in the general Greek male population [32]. The same was also observed in women COPD patients compared to the mean corresponding score (37.47) in the general Greek population of women (one-sample t test *P *< 0.01) [29]. Clinically significant levels of anxiety (score ≥ 44 for men and ≥ 46 for women) were present in 37.1% of men, and in 45.7% of women. The mean depression score was also higher than the corresponding mean score in the general population (5.86 one-sample t test *P *< 0.01) [30], while 24% and 35.3% of the sample presented mild (BDI score 10 to 14) and moderate to severe (BDI score ≥ 15) depression, respectively (Table 3).

Mean FEV_1 _percentage of predicted, age and years of education showed no correlation with alexithymia, anxiety or depression score, while strong positive correlations were noted with alexithymia, anxiety and depression (Table 4).

**Table 4:** Correlation between forced expiratory volume in 1 s (FEV_1_) percentage of predicted, depression, anxiety and alexithymia.

		FEV_1 _percentage of predicted	BDI	STAI
BDI (depression)	Pearson correlation	0.085		

	Significance (two-tailed)	0.424		

	N	167		

STAI (anxiety)	Pearson correlation	0.068	0.805*	

	Significance (two-tailed)	0.523	0.000	

	N	167	167	

TAS-20 (alexithymia)	Pearson correlation	-0.026	0.510*	0.392*

	Significance (two-tailed)	0.809	0.000	0.000

	N	167	167	167

Significant values in bold.

*Pearson correlation *P *< 0.01.

BDI = Beck Depression Inventory; STAI = Spielberger Trait Anxiety Inventory; TAS-20 = Toronto Alexithymia Scale.

To further assess factors that influence the depression score, we used stepwise multiple regression (Table 5). The trait anxiety score was responsible for 50.9% of variation in depression (F1_,165 _= 170.74, *P *< 0.001) and the alexithymia score for an additional 6.3% (F1_,164 _= 24.06, *P *< 0.01).

**Table 5:** Stepwise multiple regression (only statistically significant variables are included)*.

Variable	Multiple R	*B*	Standard error	β	t	Significance
**STAI (anxiety)**	0.713	0.445	0.041	0.606	10.908	0.000

**TAS-20 (alexithymia**)	0.756	0.182	0.037	0.273	4.905	0.000

Dependent variable: BDI (depression).

*The following variables were eliminated: age, gender, education years and FEV_1 _percentage of predicted.

BDI = Beck Depression Inventory; FEV_1 _= forced expiratory volume in 1 s; STAI = Spielberger Trait Anxiety Inventory; TAS-20 = Toronto Alexithymia Scale.

## Discussion

The present study confirms the presence of a higher proportion of anxiety (approximately 37.1% for male and 45.7% for female) and depression (approximately 35.3%) in Greek outpatients with COPD than those in the general population. However, the lack of a control group may limit the generalisability of these results. The female population with COPD is differentiated from males by higher levels of anxiety and depressive symptoms. These findings are in accordance with previous studies that indicated a higher prevalence of overall anxiety and depressive symptoms among women with COPD [15,36].

Independently of gender, there are many mechanisms that could be involved in this comorbidity. Patients with COPD have poor physical functioning, a condition which has been shown to be related to higher rate of psychological morbidity [36]. The high levels of anxiety and depressive symptoms are possibly the result of pressure from social factors, as well as from coping with daily living. Many of these patients have had to limit their daily activities due to their lung disease. They frequently have to change jobs or retire early. Their social interactions are also adversely affected because they cannot maintain pace with their peers [37]. In addition, patients with COPD soon realise that his/her disease is irreversible and progressive [14,37]. Furthermore, the hypoxic nature of the disease and dyspnoea may lead to increased distress [36,37].

However, an impressive finding of our study was that anxiety and depression were not correlated with COPD severity (as determined by FEV_1 _percentage of predicted). In a previous study [38] it was reported that dyspnoea ratings were influenced by anxiety and depressive symptoms, whereas the physiological state (including FEV_1 _percentage of predicted) scarcely influenced the anxiety and depressive symptomatology. Although further studies are required in order to explain these findings, it is possible that patients construe disease seriousness subjectively, which contributes to the development of the levels of anxiety and depressive symptoms.

The prevalence of alexithymia in COPD patients, contrary to what has been observed in patients with other chronic respiratory diseases, seems to be lower. However, a positive correlation was observed between alexithymia, anxiety and depressive symptoms. Previous studies based on both clinical and healthy populations have reported a connection between depressive symptomatology and alexithymia, and it is well known that patients with depressive disorders are prone to experiencing alexithymic features [39,40]. Additionally, alexithymic features have been related to higher levels of anxiety [41]. Due to the limitations of our study, we cannot answer the question of whether alexithymia leads to depressive and anxiety symptoms or depression and anxiety symptoms lead to alexithymia.

Compared with other psychosomatic and somatic diseases, such as bronchial asthma, the prevalence of alexithymia in COPD is lower. Furthermore, alexithymia may be related to recurrent very severe asthma exacerbations in asthmatics [42-44].

We did not study possible associations of COPD exacerbations with any of the other parameters studied. Thus, although we cannot support a similar hypothesis for COPD exacerbations, we believe that the correlations that were seen among alexithymia, depression and anxiety levels should be taken into consideration when drafting psychotherapeutic interventions (as a part of a pulmonary rehabilitation program) for these patients [45]. This is more pertinent in those patients with overall alexithymic characteristics (who often fail to recognise their underlying psychological malaise due to a lack of capacity for mental representation of emotions) [46,47]. These deficiencies may cause an inability to regulate emotions and affect and, therefore, may lead to increased somatisation and attenuated capacity to recognise the underlying depressive symptoms or anxiety (and lack thereof of therapeutic intervention) [44,47]. Furthermore, subjects with high anxiety and depressive symptoms and concomitant alexithymia most probably have difficulty in verbally expressing their symptoms [22]. In alexithymia, by definition, the difficulty in expressing psychological symptoms as such leads to their expression as somatic (often atypical) symptoms [22]. The latter may distract clinicians and make them miss the psychological component that lies at the root of the problem. Given this, the possible presence of alexithymia should be taken into consideration when planning specialised psychotherapeutic interventions within respiratory rehabilitation programs.

Additionally, patients with severe depression and anxiety are less likely to be compliant to treatment plans and more likely to be hospitalised [48]. Therefore, comprehensive programs should incorporate individualised depression and anxiety management techniques.

Finally, this study does have some limitations. First is the lack of a control group; second, we did not study possible associations of COPD exacerbations with any of the other parameters studied. These limitations should be taken into consideration in further work.

## Conclusions

This study confirms the high prevalence of anxiety and depression symptoms in Greek outpatients with COPD, a finding that is in accordance with relative studies worldwide. Additionally, our results are in agreement with other studies, which concluded that women have more psychological comorbidity [49-55].

In our study and in the most previous studies of patients with COPD, FEV_1 _percentage of predicted appeared to have an unfavourable effect, being a predictor of anxiety and depression in [50].

The prevalence of alexithymia in COPD patients, contrary to what has been observed in patients with other chronic respiratory diseases, seems to be lower. However, we observed a strong correlation between alexithymia, depression and anxiety levels, a finding suggesting that this comorbidity should be taken into consideration when drafting psychotherapeutic programs for these patients

## Competing interests

The authors declare that they have no competing interests.

## Authors' contributions

AT conceived the paper, designed the study, performed the psychological measures, collected data, carried out the statistical analysis and drafted the paper; EK performed the physical measures, carried out the statistical analysis and helped draft the paper; DB performed the psychological measures, collected data, gave suggestions for the concept of alexithymia and helped draft the paper; GM and AK helped draft the paper; II carried out the statistical analysis and helped draft the paper; NS and AV supervised the study; NT carried out the statistical analysis, helped draft the paper and supervised the study. All authors read and approved the final manuscript.
